# Evaluation of a scalable psychosocial intervention for refugees in Greece

**DOI:** 10.1192/j.eurpsy.2024.1272

**Published:** 2024-08-27

**Authors:** C. Papathanasiou, A. Kougioumtzi

**Affiliations:** ^1^Department of Psychology, Panteion University of Social and Political Sciences; ^2^Department of Refugee Care, Association for Regional Development and Mental Health, Athens, Greece

## Abstract

**Introduction:**

The world of the third millennium is witnessing the highest levels of displacement on record. To meet the specific needs of this vulnerable population, a task-shifting approach is developed, where individuals with refugee background and lived experience are trained and supervised my mental health professionals to provide emotional and practical support to members of their communities.

**Objectives:**

The evaluation of a scalable psychosocial intervention for refugees based on the task-shifting approach.

**Methods:**

The intervention consisted of sessions of Problem Management Plus (PM+) and peer case management delivered by a team of community psychosocial workers (trained refugees). The sample consisted of 173 participants, Arabic- and Farsi-speakers male and female, recognized refugees, and asylum seekers. Anxiety, depression, and psychological distress were measured before and after the intervention using the Generalised Anxiety Disorder-7 (GAD-7), Patient Health Questionnaire - 9 (PHQ-9), and Psychological Outcome Profiles (Psychlops) scales respectively. Repeated measures analysis of variance (ANOVA) was adopted to evaluate the difference in the degree of change across patients’ characteristics over the follow up period. Statistical significance was set at p<0.05 and analyses were conducted using SPSS statistical software (version 26.0).

**Results:**

Significant decreases were found in all post-test scales, indicating diminution of anxiety, depression symptoms, and psychological distress. Large effects sizes were found in all scales.

**Conclusions:**

The findings support that task-shifting approach incorporating PM+ and case management is effective for the mental health of refugees. Peer support could be included in a stepped care model for refugee mental health and well-being in high-income countries. For future research a randomized controlled trial is proposed as a study protocol.

**Disclosure of Interest:**

None Declared

